# Sparse Coding and Counting for Robust Visual Tracking

**DOI:** 10.1371/journal.pone.0168093

**Published:** 2016-12-16

**Authors:** Risheng Liu, Jing Wang, Xiaoke Shang, Yiyang Wang, Zhixun Su, Yu Cai

**Affiliations:** 1 School of Software Technology, Dalian University of Technology, Dalian City, Liaoning Province, China; 2 Key Laboratory for Ubiquitous Network and Service Software of Liaoning Province, Dalian City, Liaoning Province, China; 3 School of Mathematic Sciences, Dalian University of Technology, Dalian City, Liaoning Province, China; 4 Dalian Campus, Luxun Academy of Fine Arts, Dalian City, Liaoning Province, China; Tianjin University, CHINA

## Abstract

In this paper, we propose a novel sparse coding and counting method under Bayesian framework for visual tracking. In contrast to existing methods, the proposed method employs the combination of *L*_0_ and *L*_1_ norm to regularize the linear coefficients of incrementally updated linear basis. The sparsity constraint enables the tracker to effectively handle difficult challenges, such as occlusion or image corruption. To achieve real-time processing, we propose a fast and efficient numerical algorithm for solving the proposed model. Although it is an NP-hard problem, the proposed accelerated proximal gradient (APG) approach is guaranteed to converge to a solution quickly. Besides, we provide a closed solution of combining *L*_0_ and *L*_1_ regularized representation to obtain better sparsity. Experimental results on challenging video sequences demonstrate that the proposed method achieves state-of-the-art results both in accuracy and speed.

## Introduction

Visual tracking plays an important role in computer vision and has many applications such as video surveillance, robotics, motion analysis and human computer interaction. Even though various algorithms have come out, it is still a challenge problem due to complex object motion, heavy occlusion, illumination change and background clutter.

Visual tracking algorithms can be roughly categorized into two major categories: discriminative methods and generative methods. Discriminative methods (*e.g.*, [1–3]) view object tracking as a binary classification problem in which the goal is to separate the target object from the background. Generative methods (*e.g.*, [4–8]) employ a generative appearance model to represent the target’s appearance.

We focus on the generative one and will briefly review the relevant work below. Recently, sparse representation has been successfully applied to visual tracking (*e.g.*, [9–12]). The trackers based on sparse representation are under the assumption that the appearance of a tracked object can be sparsely represented by a over-complete dictionary which can be dynamically updated to maintain holistic appearance information. Traditionally, the over-complete dictionary is a series of redundant object templates, however, a set of basis vectors from target subspace as dictionary is also used because an orthogonal dictionary performs as efficient as the redundant one. In visual tracking, we will call the *L*_1_ regularized object representation “sparse coding” (*e.g.*, [9]), and the *L*_0_ regularized object representation “sparse counting” (*e.g.*, [13]). [9] has been shown to be robust against partial occlusions, which improves the tracking performance. However, because of using redundant dictionary, heavy computational overhead in *L*_1_ minimization hampers the tracking speed. Very recent efforts have been made to improve this method in terms of both speed and accuracy by using accelerated proximal gradient (APG) algorithm [14] or modeling the similarity between different candidates [11]. Different from [9], IVT [5] incrementally learns a low-dimensional PCA subspace representation, which adapts online to the appearance changes of the target. To get rid of image noise, Lu *et al.* [15] introduce *L*_1_ noise regularization into the PCA reconstruction, which is able to handle partial occlusion and other challenging factors. Pan *et al.* [13] employs *L*_0_ norm to regularize the linear coefficients of incrementally updated linear basis (sparse counting) to remove the redundant features of the basis vectors. However, sparse counting will cause unstable solutions because of its nonconvexity and discontinuity. Although the sparse coding has good performance, it may cause biased estimation since it penalizes true large coefficients more, and produce over-penalization. Consequently, it is necessary to find a way to overcome the disadvantages of spare coding and sparse counting.

From the viewpoint of statistics, sparse representation are similar to variable selection when the dictionary is fixed. Besides, it is a bonus that Bayesian framework has been successfully applied to select variables by enforcing appropriate priors. Laplace priors were used to avoid overfitting and enforce sparsity in sparse linear model, which derives sparse coding problem. To further enforce sparsity and reduce over-penalization of sparse coding, each coefficient is assigned with a Bernoulli variable. Therefore, a novel model interpreted from a Bayesian perspective by carrying maximum a posteriori (MAP) is proposed, which turns out to be a combination of sparse coding and counting model. In paper [16], Lu *et al.* also consider *L*_0_ and *L*_1_ norm under a Bayesian perspective. However, considering that there will be occlusion, illumination change and background clutter in tracking, we restraint the noise with *L*_1_ norm. Besides, We use an orthogonal dictionary to replace the redundant object templates as similar atoms of redundant templates may cause mistake of coefficients and huge computational complexity. Lastly, We propose closed solution of regularization which is the combination of the *L*_0_ norm and *L*_1_ norm. However Lu *et al.* obtain the approximate solution by using he Greedy Coordinate Descent.

**Fig 1:**
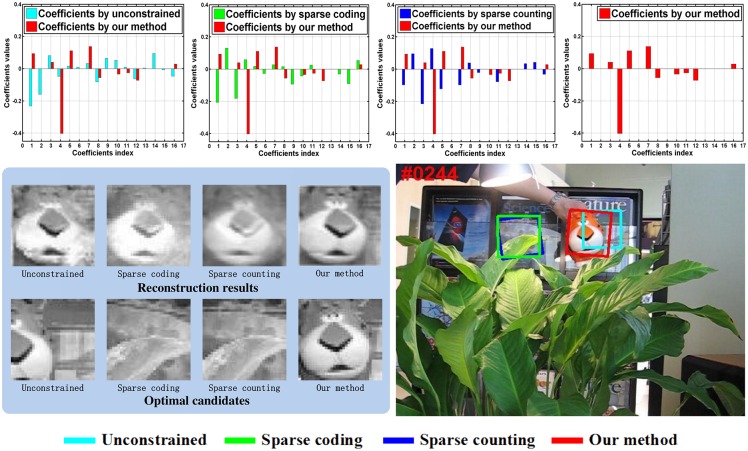
The comparison of coefficients, optimal candidates and reconstruction. The top is the coefficients of our method versus unconstrained, spars coding and sparse counting regularization, respectively. The bottom is the optimal candidates and reconstruction results by using unconstrained, sparse coding, sparse counting and our method under same dictionary, respectively.

**Fig 2:**
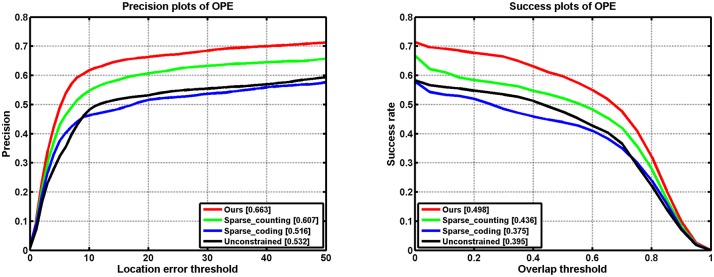
Precision and success plots of overall performance comparison among unconstrained regularization, sparse counting, sparse coding and ours for the 22 videos in the benchmark. The mean precision scores are reported in the legends.

Tracking results by using unconstrained regularization, sparse counting, sparse coding and our model under the same dictionary *D* are shown in Fig 1, respectively. As shown in Fig 1, one can see that the coefficients of unconstrained regularization and sparse coding are actually not sparse and the target object is not tracked well. Similarly, sparse counting with sparsity coefficients sometimes cannot obtain appropriate linear combination of the orthogonal basis vectors, which will interfere with the tracking accuracy. However, we note that our method is able to reconstruct the object well and find the good candidate, then facilitating the tracking results. We also compare our model with unconstrained regularization, sparse counting, sparse coding over all 50 sequences in benchmark, the precision and success plots are shown in Fig 2. One can see the parameter setting in the section Experimental Results.

**Contributions**: The contributions of this work are threefold.

- We propose a sparse coding and counting model from a novel Bayesian perspective for visual tracking. Compared to the state-of-the-art algorithms, the proposed method achieves more reliable tracking results.
- We propose closed solution of combining the *L*_0_ norm and *L*_1_ norm based regularization in a unique one.
- Although the sparse coding and counting related minimization is an NP-hard problem, we show that the proposed model can be efficiently estimated by the proposed APG method. This makes our tracking method computationally attractive in general and comparable in speed with SP method [15] and the accelerated *L*_1_ tracker [14].

## Visual Tracking based on the Particle Filter

In this paper, we employ a particle filter to track the target object. The particle filter provides an estimate of posterior distribution of random variables related to Markov chain. Given a set of observed image vectors **Y**_*t*_ = {**y**_1_, **y**_2_, …, **y**_*t*_} up to the *t*-th frame and target state variable **x**_*t*_ that describes the six affine motion parameters, the posterior distribution *p*(**x**_*t*_|**Y**_*t*_) based on the Bayesian theorem is estimated by:

$$\begin{array}{c} p\left(x_{t}|Y_{t}\right)\propto p\left(y_{t}|x_{t}\right)\int p\left(x_{t}|x_{t-1}\right)p\left(x_{t-1}|Y_{t-1}\right)dx_{t-1}, \end{array}$$ (1)

where *p*(**y**_*t*_|**x**_*t*_) is the observation model that estimates the likelihood of an observed image patch **y**_*t*_ belonging to the object class, and *p*(**x**_*t*_|**x**_*t*−1_) is the motion model that describes the state transition between consecutive frames.

**The Motion Model:** The motion model *p*(**x**_*t*_|**x**_*t*−1_) = *N*(**x**_*t*_; **x**_*t*−1_, **Σ**) models the parameters by independent Gaussian distribution around the counterpart in **x**_*t*−1_, where **Σ** is a diagonal covariance matrix whose elements are the variances of the affine parameters. In the tracking framework, the optimal target state ${\hat{x}}_{t}$ is obtained by the maximal approximate posterior (MAP) probability: ${\hat{x}}_{t}=\arg\max_{x_{t}^{i}}p\left(x_{t}^{i}|Y_{t}\right)$, where $x_{t}^{i}$ indicates the *i*-th sample of the state **x**_*t*_.

**The observation model:** In this paper, we assume that the tracked target object is generated by a subspace (spanned by **D** and centered at ***μ***) with corruption (i.i.d Gaussian Laplacian noise),

$$\begin{array}{c} y=D\alpha +\epsilon +e, \end{array}$$ (2)

where $y\in R^{N}$ denotes an observation vector centered at ***μ***, the columns of $D=\left\{d_{1},d_{2},\ldots ,d_{K}\right\}\in R^{N\times K}$ are orthogonal basis vectors of the subspace, ***α*** indicates the coefficients of basis vectors, **ϵ** and **e** stand for the Gaussian noise and Laplacian noise vector respectively. the Gaussian component models small dense noise and the Laplacian one aims to handle outliers. As proposed by [17], under the i.i.d Gaussian-Laplacian noise assumption, the distance between the vector **y** and the subspace (**D**, ***μ***) is the least soft threshold squares distance:

$$\begin{array}{c} d\left(\alpha ,e\right)=\min_{\alpha ,e}\frac{1}{2}{∥y-D\alpha -e∥}_{2}^{2}+\lambda {∥e∥}_{1}. \end{array}$$ (3)

Thus, for each observation **y**_*t*_ corresponding to a predicted state **x**_*t*_, the observation model *p*(**y**_*t*_|**x**_*t*_) that is set to be

$$\begin{array}{c} p\left(y_{t}|x_{t}\right)=\exp\left(-\tau d\left(\alpha^{*},e^{*}\right)\right), \end{array}$$ (4)

where α* and e* are the optimal solution of Eq (18) which will be introduced in detail in next section, and *τ* is a constant controlling the shape of the Gaussian kernel.

**Model Update:** It is essential to update the observation model for handling appearance change of the target in visual tracking. Since the error term **e** can be used to identify some outliers (*e.g.*, Laplacian noise, illumination), we adopt the strategy proposed by [17] to update the appearance model using the incremental PCA with mean update [5] as follows,

$$\begin{array}{c} y_{i}=\left\{ \begin{array}{cc} y_{i}, & e_{i}=0,\\ \mu_{i}, & \;\text{otherwise}, \end{array}\right. \end{array}$$ (5)

where *y*_*i*_, *e*_*i*_, and *μ*_*i*_ are the i-th elements of **y**, **e**, and ***μ***, respectively, ***μ*** is the mean vector computed the same as [5].

## Object Representation by Bayesian Framework

### Motivation

Considering **y** as the vectorized target object region, it can be represented by an feature subspace with both sparse corruptions and dense errors, i.e.,

$$\begin{array}{c} y=D\alpha +\epsilon +e. \end{array}$$ (6)

Most existing sparsity based trackers aim to directly utilize *L*_1_ regularization on ***α*** to suppress small coefficients for subspace reconstruction. However, by carefully investigating the soft-thresholding operator corresponding to *L*_1_ minimization subproblem, it can be observed that such simple regularization will consistently suppress the values of the coefficients, thus destroy the discriminative property of the learned feature subspace.

To address this limitation in existing work, we here incorporate two different sparse regularization techniques within the Bayesian perspective, which has the capacity to encode prior knowledge and to make valid estimation of uncertainty. In other words, our goal is to propose a Bayesian inference framework to incorporate both the coefficients threshoding and selection to improve the discrimination of our feature subspace learning formulation Specifically, by defining an index vector **r** = [*r*_1_, *r*_2_, …, *r*_*K*_] ($r_{l}=I\left(\alpha_{l}\neq 0\right),l=1,2,\ldots ,K$), Eq (6) can be rewritten as

$$\begin{array}{c} y_{j}=\sum_{l=1}^{K}d_{jl}r_{l}\alpha_{l}+\epsilon_{j}+e_{j},\;j=1,2,\ldots ,N. \end{array}$$ (7)

Here the additional index vector **r** can be considered as a dictionary selection operator and we will enforce particular prior distribution on it to enhance the discriminative power of our model for subspace reconstruction. To further enhance the representative ability of our model, we will also develop a novel dictionary learning framework to build orthogonal subspace dictionary for Eq (6). Please notice that the orthogonality of the learned dictionary will also significantly simply the numerical optimization process. Please see the following sections for more details.

### Bayesian Formulation

Now we will introduce our model under Bayesian framework in detail. The joint posterior distribution of ***α***, **r**, **e** and *σ*^2^ based on the Bayesian theorem can be written as

$$\begin{array}{ccc} & & p(\alpha ,r,e,\sigma^{2}|D,y,\tilde{\mu },\tau_{1},\tau_{2},\kappa ,\hat{\sigma })\propto \\ & & \;\;p\left(y|,D,\alpha ,r,e,\sigma^{2}\right)p\left(\alpha |\sigma^{2},\tilde{\mu }\right)p\left(r|\kappa \right)p\left(e|\hat{\sigma }\right)p\left(\sigma^{2}|\tau_{1},\tau_{2}\right), \end{array}$$ (8)

where *p*(**y**|**D**, ***α***, **r**, **e**, *σ*^2^), $p(\alpha |\sigma^{2},\tilde{\mu })$, *p*(**r**|*κ*), $p(e|\hat{\sigma })$, *p*(*σ*^2^|*τ*_1_, *τ*_2_), denote the priors on the noisy vectorized target region, the coefficient vector ***α*** = [*α*_1_, *α*_2_, …, *α*_*K*_], the index vector **r** = [*r*_1_, *r*_2_, …, *r*_*K*_] ($r_{l}=I\left(\alpha_{l}\neq 0\right),l=1,2,\ldots ,K$), the Laplacian noise, and the noise level, respectively. In Eq (8), the parameters $\tilde{\mu }$, *τ*_1_, *τ*_2_, $\hat{\sigma }$, and *κ* are the relevant constant parameters of the priors.

We generally assume that the noise *ϵ*_*j*_ follows the Gaussian distribution, *i.e.*, *p*(*ϵ*_*j*_) = *N*(0, *σ*^2^). We treat the Laplacian noise term *e*_*j*_ as missing values with the same Laplacian prior. Therefore, the Prior *p*(**y**|, **D**, ***α***, **r**, **e**, *σ*^2^) has the follow distribution:

$$\begin{array}{ccc} & & p(y|,D,\alpha ,r,e,\sigma^{2})\\ & & \;\;=\prod_{j=1}^{N}P\left(y_{j}|,d_{j},\alpha ,r,e_{j},\sigma^{2}\right)=\prod_{j=1}^{N}N\left(\sum_{l=1}^{K}d_{jl}r_{l}\alpha_{l}+e_{j},\sigma^{2}\right). \end{array}$$ (9)

To enforce sparsity, the coefficients ***α*** are assumed to follow Laplace distribution.

$$\begin{array}{c} p\left(\alpha |\sigma^{2},\tilde{\mu }\right)=\prod_{l=1}^{K}p\left(\alpha_{l}|\sigma^{2},\tilde{\mu }\right)=\prod_{l=1}^{K}\frac{1}{2\sigma^{2}{\tilde{\mu }}^{-1}}\exp\left(-\frac{|\alpha_{l}|}{\sigma^{2}{\tilde{\mu }}^{-1}}\right). \end{array}$$ (10)

Our goal is to remove redundant features while preserving the useful parts in the dictionary. As Laplace priors resulting sparse coding may lead to over penalization on the large coefficients, we assume the index variable *r*_*l*_ of each coefficient *α*_*l*_ to be a Bernoulli variable to enforce sparsity and reduce over penalization.

$$\begin{array}{c} p\left(r|\kappa \right)=\prod_{l=1}^{K}\kappa^{r_{l}}{\left(1-\kappa \right)}^{1-r_{l}}, \end{array}$$ (11)

where *κ* ≤ 1/2. Here, the Bernoulli prior on *r*_*l*_ means that *r*_*l*_ will have probability *κ* to be 1 and 1 − *κ* to be 0, if the prior information is known.

The noise *e*_*j*_ is aims at handling outliers, so it follows Laplace distribution:

$$\begin{array}{c} p\left(e|\hat{\sigma }\right)=\prod_{j=1}^{N}p\left(e_{j}|\hat{\sigma }\right)=\prod_{j=1}^{N}\frac{1}{2\hat{\sigma }}\exp\left(-\frac{|e_{j}|}{\hat{\sigma }}\right). \end{array}$$ (12)

The variances of noises are assigned with Inverse Gamma prior as follow:

$$\begin{array}{c} p\left(\sigma^{2}|\tau_{1},\tau_{2}\right)=\frac{\tau_{2}^{\tau_{1}}}{\Gamma (\tau_{1})}\sigma^{-2(\tau_{1}+1)}\exp\left(-\frac{\tau_{2}}{\sigma^{2}}\right), \end{array}$$ (13)

where Γ(⋅) denotes the gamma function.

Then, the optimal ***α***, **r**, **e**, *σ*^2^ are obtained by the MAP probability. After taking the negative logarithm, the formula is

$$\begin{array}{c} \left(\alpha^{*},r^{*},e^{*},\sigma^{*2}\right)={\arg\min}_{\alpha ,r,e,\sigma^{2}}\left\{-2\log p\left(\alpha ,r,e,\sigma^{2}|D,y,\tilde{\mu },\tau_{1},\tau_{2},\kappa ,\hat{\sigma }\right)\right\}. \end{array}$$ (14)

Combining the aforementioned Eqs (8)–(13), we have

$$\begin{array}{ccc} & & -2\log p(\alpha ,r,e,\sigma^{2}|D,y,\tilde{\mu },\tau_{1},\tau_{2},\kappa ,\hat{\sigma })\\ & & \;\;=\frac{1}{\sigma^{2}}\sum_{j=1}^{N}{\left(y_{j}-\sum_{l=1}^{K}d_{jl}r_{l}\alpha_{l}-e_{j}\right)}^{2}+\frac{1}{\sigma^{2}}\frac{2\sigma^{2}}{\hat{\sigma }}\sum_{j=1}^{N}|e_{j}|+\frac{2\tilde{\mu }}{\sigma^{2}}\sum_{l=1}^{K}\left|\alpha_{l}\right|+\\ & & \;\;\left(2N+2K+2\tau_{1}+2\right)\log\sigma^{2}+\frac{2\tau_{2}}{\sigma^{2}}+\sum_{l=1}^{K}r_{l}\log\frac{{\left(1-\kappa \right)}^{2}}{\kappa^{2}}+const. \end{array}$$ (15)

With fixing *σ*^2^ = 1, Eq (15) can be rewritten as

$$\begin{array}{c} {\left||y-D\alpha -e|\right|}_{2}^{2}+2\beta {\left||e|\right|}_{1}+2\tilde{\mu }{\left||\alpha |\right|}_{1}+\rho_{\kappa }{\left||\alpha |\right|}_{0}+const, \end{array}$$ (16)

where $\rho_{\kappa }=\log{\left(1-\kappa \right)}^{2}/\kappa^{2},\beta =\sigma^{2}/\hat{\sigma }$. With $\gamma \in \left[0,1\right],\lambda =\tilde{\mu }+1/2\rho_{\kappa }$ and $\gamma =4\tilde{\mu }/\left(2\tilde{\mu }+\rho_{\kappa }\right)$, Eq (16) can be rewritten as

$$\begin{array}{c} \frac{1}{2}{\left||y-D\alpha -e|\right|}_{2}^{2}+\beta {\left||e|\right|}_{1}+\lambda (\gamma {\left||\alpha |\right|}_{1}+\left(1-\gamma \right){\left||\alpha |\right|}_{0})+const. \end{array}$$ (17)

### Final Optimization Model

By observing the objective function in Eq (17), it can be found that the essential regularization in Eq (17) is a combination of the sparse coding and the sparse counting. With a fixed appropriate orthogonal dictionary D, Eq (17) can be written as the following optimization problem

$$\begin{array}{c} \min_{\alpha \;e}\frac{1}{2}{\left||y-D\alpha -e|\right|}_{2}^{2}+\beta {\left||e|\right|}_{1}+\lambda (\gamma {\left||\alpha |\right|}_{1}+\left(1-\gamma \right){\left||\alpha |\right|}_{0}), \end{array}$$ (18)

where ‖⋅‖_0_ denotes the *L*_0_ norm which counts the number of non-zero elements, *γ*, *λ* and *β* are regularization parameters, and ‖⋅‖_2_ and ‖ ⋅ ‖_1_ denote *L*_2_ and *L*_1_ norms, respectively. The term ‖**e**‖_1_ is used to reject outliers (*e.g.*, occlusions), while ‖***α***‖_0_ and ‖***α***‖_1_ are used to select the most discriminative subspace features. Notice that we also implicitly assume that **D**^⊤^ **D** = **I**, where **I** is an identity matrix.

## Theory of Fast Numerical Algorithm

It is known that APG is an excellent algorithm for convex programming [18, 19] and has been used in visual tracking. In this section, we propose a fast numerical algorithm for solving the proposed nonconvex and nonsmooth model by using APG approach. The experimental results show that it can converge to a solution quickly and achieve attractive performance. Besides, the closed solution of the combining *L*_0_ and *L*_1_ based regularization is provided.

### APG Algorithm for Solving Eq (19)

Eq (18) contains two subproblem: one is solving ***α*** given fixed **e**, the other one is solving **e** given fixed ***α***, the formula is shown as follow

$$\begin{array}{c} \left\{ \begin{array}{c} \alpha =\arg\min_{\alpha }\frac{1}{2}{\left||y-D\alpha -e|\right|}_{2}^{2}+\lambda \gamma {\left||\alpha |\right|}_{1}+\lambda \left(1-\gamma \right){\left||\alpha |\right|}_{0},\\ e=\arg\min_{e}\frac{1}{2}{\left||y-D\alpha -e|\right|}_{2}^{2}+\beta {\left||e|\right|}_{1}. \end{array}\right. \end{array}$$ (19)

Solving Eq (19) is an NP-hard problem because it involves a discrete counting metric. We adopt a special optimization strategy based on the APG approach [18], which ensures each step be solved easily. In APG Algorithm, we need to solve

$$\begin{array}{c} \left\{ \begin{array}{c} \alpha_{k+1}^{*}=\arg\min_{\alpha }\lambda \gamma {∥\alpha ∥}_{1}+\lambda \left(1-\gamma \right){∥\alpha ∥}_{0}+\frac{L}{2}{∥\alpha -z_{k+1}^{\alpha }+\frac{\nabla_{\alpha }F\left(z_{k+1}\right)}{L}∥}_{2}^{2},\\ e_{k+1}^{*}=\arg\min_{e}\beta {∥e∥}_{1}+\frac{L}{2}{∥e-z_{k+1}^{e}+\frac{\nabla_{e}F\left(z_{k+1}\right)}{L}∥}_{2}^{2}, \end{array}\right. \end{array}$$ (20)

where $z_{k+1}=\left(z_{k+1}^{\alpha },z_{k+1}^{e}\right)$, ∇_***α***_ *F*(***α***, **e**) = **D**^⊤^(**D*α*** + **e** − **y**), ∇_**e**_ *F*(***α***, **e**) = **e** − (**y** − **D** ***α***), and *L* is a Lipschitz constant.

The solutions of Eq (20) can be obtained by

$$\begin{array}{c} \left\{ \begin{array}{c} \alpha_{k+1}^{*}=E_{\left(\lambda \gamma /L,\lambda (1-\gamma )/L\right)}(z_{k+1}^{\alpha }-\frac{\nabla_{\alpha }F\left(z_{k+1}\right)}{L}),\\ e_{k+1}^{*}=S_{\beta /L}(z_{k+1}^{e}-\frac{\nabla_{e}F\left(z_{k+1}\right)}{L}), \end{array}\right. \end{array}$$ (21)

where $S_{\theta }\left(y\right)=\text{sign}\left(y\right)\max\left(|y|-\theta ,0\right)$, and $E_{\left(\delta ,\eta \right)}\left(y\right)$ is defined as

$$\begin{array}{c} E_{\left(\delta ,\eta \right)}\left(y\right)=\left\{ \begin{array}{cc} y-\delta , & y>\delta +\sqrt{2\eta },\\ y+\delta , & y<-\delta -\sqrt{2\eta },\\ 0, & otherwise. \end{array}\right. \end{array}$$ (22)

The numerical algorithm for solving Eq (19) is summarized in Algorithm 1. Due to the orthogonality of **D**, Algorithm 1 converges fast, and its computation cost does not increase compared to the solver of *L*_1_ regularized model.

**Algorithm 1** Fast Numerical Algorithm for Solving Eq (19)

**Initialize:** Set initial guesses ***α***_0_ = ***α***_−1_ = **0**, **e**_0_ = **e**_−1_ = **0**, and *t*_0_ = *t*_−1_ = 1.

**while** not convergence or termination **do**

**Step 1:** $z_{k+1}^{\alpha }:=\alpha_{k}+\frac{t_{k-1}-1}{t_{k}}\left(\alpha_{k}-\alpha_{k-1}\right)$;

**Step 2:** $z_{k+1}^{e}:=e_{k}+\frac{t_{k-1}-1}{t_{k}}\left(e_{k}-e_{k-1}\right)$;

**Step 3:** $\alpha_{k+1}=E_{\left(\lambda \gamma /L,\lambda (1-\gamma )/L\right)}(z_{k+1}^{\alpha }-\frac{\nabla_{\alpha }F\left(z_{k+1}\right)}{L})$;

**Step 4:** $e_{k+1}=S_{\beta /L}(z_{k+1}^{e}-\frac{\nabla_{e}F\left(z_{k+1}\right)}{L})$;

**Step 5:** $t_{k+1}:=\frac{1+\sqrt{1+4t_{k}^{2}}}{2}$, *k* ← *k*+1.

**end while**

### Closed-form Solution for Combining *L*_1_ and *L*_0_ Regularization

This subsection mainly focus on a sparse combinatory model which combines *L*_0_ and *L*_1_ norm together as the regularizer term

$$\begin{array}{c} \min_{x}\frac{1}{2}{\left(x-y\right)}^{2}+\delta \left|x\right|+\eta {\left|x\right|}_{0}, \end{array}$$ (23)

where $x,y\in R^{1}$, and |*x*| denotes *L*_0_ norm: if *x* = 0, then |*x*|_0_ = 0, and |*x*|_0_ = 1, otherwise.

**Proposition 1.** *The optimal solution* x* *of the* Eq (23) *is defined as*

$$\begin{array}{c} x^{*}=\left\{ \begin{array}{cc} y-\delta , & y>\delta +\sqrt{2\eta },\\ y+\delta , & y<-\delta -\sqrt{2\eta },\\ 0, & otherwise. \end{array}\right. \end{array}$$ (24)

*Proof*. First, we denote $E\left(x\right)=\frac{1}{2}{\left(x-y\right)}^{2}+\delta \left|x\right|+\eta {\left|x\right|}_{0}$. It is obvious that if *x* = 0, then $E\left(0\right)=\frac{1}{2}y^{2}$. Then we need to discuss the case that *x* ≠ 0:

1. if *x* > 0, then $E\left(x\right)=\frac{1}{2}{\left(x-y\right)}^{2}+\delta x+\eta$. Writing its K.K.T condition, we get *x* = *y* − *δ*, and the objective value is $E\left(y-\delta \right)=-\frac{1}{2}\delta^{2}+\delta y+\eta$.
2. if *x* < 0, then $E\left(x\right)=\frac{1}{2}{\left(x-y\right)}^{2}-\delta x+\eta$. It is easy to get *x* = *y* + *δ*, and the objective value is $E\left(y+\delta \right)=-\frac{1}{2}\delta^{2}-\delta y+\eta$.

Then, we need to compare these three cases, if *E*(0) > *E*(*x* − *δ*), we have (*δ* − *y*)^2^ > 2*η*. Combining with *x* = *y* − *δ* > 0, we have $y>\delta +\sqrt{2\eta }$. Similarly, if *E*(0) > *E*(*x* + *δ*), then we have $y<-\delta -\sqrt{2\eta }$. And *x* = 0, otherwise.

If $x\in R^{N}$, the Eq (23) changes into

$$\begin{array}{c} \min_{x}\frac{1}{2}{\left||x-y|\right|}_{2}^{2}+\delta {\left||x|\right|}_{1}+\eta {\left||x|\right|}_{0}, \end{array}$$ (25)

where ${\left||x|\right|}_{1}=\sum_{i=1}^{N}\left|x_{i}\right|$ and ${\left||x|\right|}_{0}=\sum_{i=1}^{N}{|x_{i}|}_{0}$. It is obvious that Eq (23) can be turned into

$$\begin{array}{c} \min_{x_{i}}\sum_{i=1}^{N}\frac{1}{2}{\left(x_{i}-y_{i}\right)}^{2}+\delta \left|x_{i}\right|+\eta {|x_{i}|}_{0}. \end{array}$$ (26)

So it can be seen as a sequence of optimization of *x*_*i*_, *i* = 1, …, *n*, and each can be solved by proposition.

**Fig 3:**
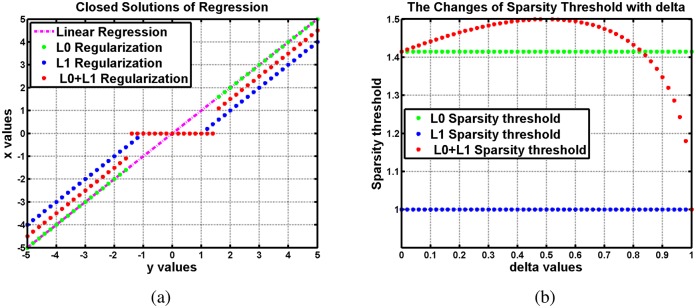
Analysis about combination of *L*_1_ and *L*_0_ regularization. (a) shows the closed solutions of linear regression, *L*_0_, *L*_1_, *L*_0_ + *L*_1_ regularized regression, respectively. (b) shows the sparsity threshold changes of *L*_0_, *L*_1_ and *L*_0_ + *L*_1_ regularized regression, respectively.

In Eq (25), if we set *δ* = 0 and *η* = 0, the model degenerates to the linear regression. If we set *δ* = 0, Eq (25) reduces to *L*_0_ regularized regression, while becoming *L*_1_ regularized regression when *η* = 0. Fig 3(a) shows the closed solutions of these four cases. We set *δ* = *η* = 0.5 in Eq (25) (*L*_0_ + *L*_1_ regularized regression), *η* = 1 in *L*_0_ regularized regression, and *δ* = 1 in *L*_1_ regularized regression. We note that *L*_0_ + *L*_1_ regularized regression has the same sparsity as *L*_0_ regularized regression, while causing little over penalization than *L*_1_ regularized regression. In Fig 3(b), sparsity threshold changes of *L*_0_, *L*_1_ and *L*_0_ + *L*_1_ regularized regression are shown, respectively. When *δ* = 1 − *η* changes from 0 to 1, the sparsity threshold of *L*_0_ + *L*_1_ varies from that of *L*_0_ to the threshold of *L*_1_. Besides, it is obvious that the threshold of *L*_0_ + *L*_1_ is larger than those of *L*_0_ and *L*_1_ in interval (0, 0.8].

## Orthogonal Dictionary Learning for Visual Tracking

In this section, we demonstrate dictionary learning in detail through three parts: dictioanry initialization, orthogonal dictionary update and dictionary reinitialization.

**Dictioanry Initialization:** There are two schemes to initialize the orthogonal dictionary, one is doing PCA for the set of initial first *k* frames **Y**_*k*_, the other is doing RPCA for **Y**_*k*_. When initial frames do not undergo corruption (*e.g.*, occlusion or illumination), we do PCA for **Y**_*k*_ instead of RPCA. The whole process of PCA is doing skinny SVD for **Y**_*k*_ and get the basis vectors of column space as the initial dictionary. However, when initial frames have large sparse noise, RPCA is selected to get the intrinsic low-rank features **Z**_*k*_, which can be obtained by solving [7]:

$$\begin{array}{c} \min_{Z_{k},E_{k}}{∥Z_{k}∥}_{*}+\lambda {∥E_{k}∥}_{1},\;s.t.\;Y_{k}=Z_{k}+E_{k}. \end{array}$$ (27)

**Fig 4:**
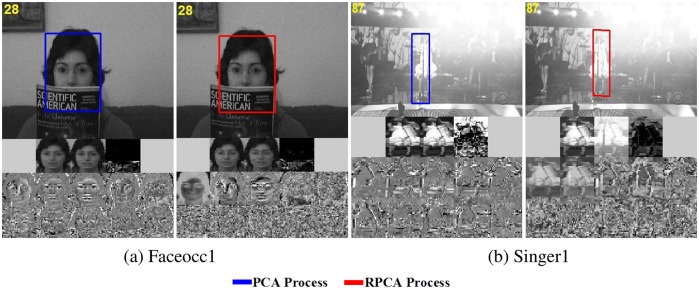
Comparison of PCA and RPCA. The upper portion of the image is the tracking frame. the middle of the image consists of three sub-pictures, the left is the mean image, the middle is the reconstruction result, and the right is the Lapalace noise. the bottom of the image is the top ten basis vectors of dictionary. (a) shows the tracking results of PCA and RPCA dictionary initialization. The tracking performance with and without RPCA reinitialization is shown in (b). Reprinted from [20] under a CC BY license, with permission from Yi Wu, original copyright 2013.

When solving Eq (27), the skinny SVD of **Z**_*k*_ is readily available: $Z_{k}=U_{k}\Sigma_{k}V_{k}^{T}$, and **D** = **U**_*k*_ is the initial orthogonal dictionary. As the analysis in [6], the skinny SVD of **Z**_*k*_ is readily available when solving Eq (27): Fig 4(a) shows that PCA initialization and RPCA initialization both perform well when the initial first *k* frames have little noise. The initial frames is generally clean, therefore, we choose PCA initialization as the default.

**Orthogonal Dictionary Update:** As the appearance of a target may change drastically, it is necessary to update the orthogonal dictionary **D**. Here we adopt an incremental PCA algorithm [21] to update the dictionary.

**Dictionary Reinitialization:** When the tracker is prone to drift, dynamically reinitializing dictionary to obtain the intrinsic subspace features is needed. We adopt the strategy proposed by [7]. The reinitialization is performed at *t*-th frame if *σ* = ‖**e**_*t*_‖_0_/*len*(**e**_*t*_) > *thr*, where **e**_*t*_ is the noise item at *t*-th frame, *len*(.) is the length of vector, and *thr* > 0 is a threshold parameter (generally 0.5). If *σ* > *thr*, we reinitialize the dictionary in the same way as initialization of dictionary by doing RPCA, but **Y**_*t*_ in Eq (27) is different. Here, **Y**_*t*_ consists of optimal candidate observations respectively from the initial *n* (generally 10) frames and the latest *t* − *n* frames (we set *t* = 30). Fig 4(b) compares the tracking performance within and without RPCA reinitialization when the object undergoes variable illumination. After reinitializing dictionary, our tracker retracks the object, so reinitializing dictionary is efficient to improve the reconstruction ability. In Algorithm 2, we summarize the overall tracking process for frame *t*.

## Experimental Results

In this section, we compare the performance of our proposed tracker with several state-of-the-art tracking algorithms, such as TLD [22], IVT [5], ASLA [23], *L*_1_APG [14], MTT [11], SP [15], SPOT [24], FOT [25], SST [26], SCM [27], MIL [2], and Struck [3], on twenty-two video sequences from the popular benchmark [20] including basketball, bolt, boy, car4, carDark, carScale, crossing, david, david2, david3, deer, faceocc1, faceocc2, fish, football, mountainBike, shaking, skating1, trellis, walking, walking2 and woman. These sequences are publicly available online at http://cvlab.hanyang.ac.kr/tracker_benchmark/datasets.html. Representative videos including Tiger1 and Singer1 have been downloaded from the open video data-sets of the paper [28]. Our tracker is implemented in MATLAB and runs at 4.2 fps on an Intel 2.53 GHz Dual-Core CPU with 8GB memory, running Windows 7 and Matlab (R2013b). We empirically set *η* = 0.1, *λ* = 0.5, *γ* = 0.1, *τ* = 0.05 and the Lipschitz constant *L* = 2. Before solving Eq (18), all the candidates **y** are centralized. Considering the efficiency, the updated orthogonal dictionary **D** is taken columns corresponding to the 16 largest eigenvalues of PCA or RPCA, 600 particles are adopted, and the model is incrementally updated every 5 frames. In the following, we present both qualitative and quantitative comparisons of above mentioned methods.

**Algorithm 2** Our Robust Visual Tracking Algorithm

**Initialization:** Initialize orthogonal dictionary **D** by performing PCA on $Y_{k}$.

**Input:** State **x**_*t*−1_ (*t* > *k*) and orthogonal dictionary **D**.

**Step 1:** Draw new samples $x_{t}^{i}$ from **x**_*t*−1_ and obtain corresponding candidates $y_{t}^{i}$.

**Step 2:** Obtain $\alpha_{t}^{i}$ and $e_{t}^{i}$ using Eq (19).

**Step 3:** For each candidate, calculate the observation probability $p(y_{t}^{i}|x_{t}^{i})$ using Eq (4).

**Step 4:** Find the tracking result patch $y_{t}^{*}$ with the maximal observation likelihood and its corresponding noise $e_{t}^{*}$.

**Step 5:** perform an incremental PCA algorithm to update the orthogonal dictionary **D** every five frames. If *σ* > *thr*, reinitializing Dictionary at *t*-th frame using Eq (27).

**Output:** State $x_{t}^{*}$ and corresponding image patch; orthogonal dictionary **D**.

### Qualitative Evaluation

**Fig 5:**
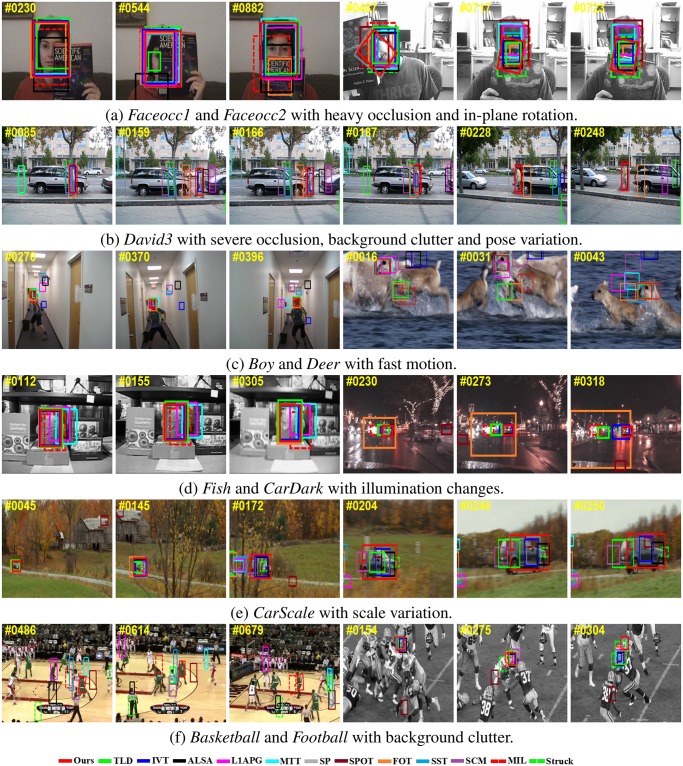
Sampled tracking results of evaluated algorithms on ten challenging image sequences.

We choose some examples from part of 22 sequences to illustrate the effectiveness of our method. Fig 5 shows the visualization results.

**Heavy Occlusion:** Fig 5(a) and 5(b) show three challenging sequences with heavy occlusion. In *Faceocc1* and *Faceocc2*, the targets undergo with heavy occlusion and in-plane rotation, it can be seen that our method outperforms the other tracking algorithms. *David3* demonstrates that the proposed method can capture the accurate location of objects in terms of position, and scale when the target undergoes severe occlusion (*e.g.*, *David3* #0085). However, IVT, *L*_1_APG, MIL, SP, SCM, ASLA, TLD, SPOT, FOT, SST, MTT, and Struck methods drift away from the target object when occlusion occurs. For these four sequences, the IVT method performs poorly since conventional PCA is not robust to occlusions. Although *L*_1_APG and SP utilize sparsity to model outliers, it is observed that their occlusion detection are not stable when drastic change of appearance happens. In contrast, our method is robust to heavy occlusion. This is because our combination of *L*_0_ and *L*_1_ regularized appearance model can exactly reconstruct the object.

**Fast Motion:** Fig 5(c) show the sequences *Boy* and *Deer* with fast motion. It is difficult to predict the locations of the tracked objects when they undergo abrupt motion. In *Boy*, the captured images are blurred seriously, but Struck and our method track the target faithfully throughout the images. IVT, MTT, ALSA, SCM and SST methods drift away seriously. We note that most of the other trackers have drift problem due to the abrupt motion and background clutter in sequence *Deer*. In contrast, the SST and our method successfully track the target for whole video.

**Illumination Changes and Scale Variation:** In Fig 5(d) and 5(e), we test three challenging sequences with illumination changes and scale variation. *Fish* chips contain significant illumination variation. We can see that the *L*_1_APG, MTT, and MIL methods are less effective in these cases (*e.g.*, *Fish* #0305). In *CarDark*, our method still performs well, but TLD, FOT, and MIL fail. Our method also achieves good performance in *CarScale* with scale variation (*e.g.*, *CarScale* #0204). For subspace-based approaches, they may fail to update the appearance model as the calculation of coefficients in their models may have redundant background features. Our method can successfully adapt to variable drastic changes since the combination of sparse coding and sparse counting is not merely stable but also applicable to obtain the intrinsic features of the subspace.

**Background Clutters:** Fig 5(f) demonstrates the tracking results in *Baskerball* and *Football* with background clutter. *Baskerball* is a difficult sequence because it contains cluttered background, illumination change, heavy occlusion and non-rigid pose variation. Unless our tracker, none of the compared algorithms can work well on it(*e.g.*, *Baskerball* #0486 and #0614). As shown in *Football*, our tracker performs relatively well (*e.g.*, *Football* #304) as it has excluded background clutters in the sparse errors, but TLD, FOT, and MIL fail.

### Quantitative Evaluation

We use two metrics to evaluate the proposed algorithm with other state-of-the-art methods. The first metric is the center location error measured with manually labeled ground truth data. The second one is the overlap rate, i.e., $score=\frac{area(R_{T}⋂R_{G})}{area(R_{T}⋃R_{G})}$, where *R*_*T*_ is the tracking bounding box and *R*_*G*_ is the ground truth bounding box. The larger average scores mean more accurate results.

**Fig 6:**
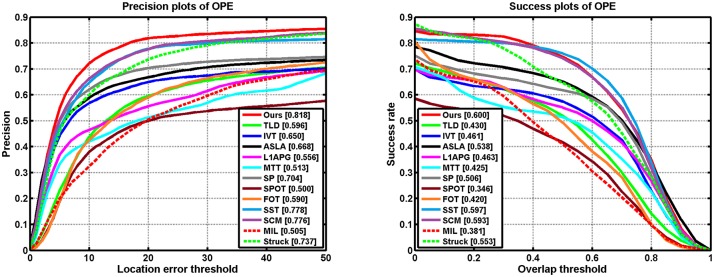
Precision and success plots over all the 22 sequences. The mean precision scores are reported in the legends.

**Fig 7:**
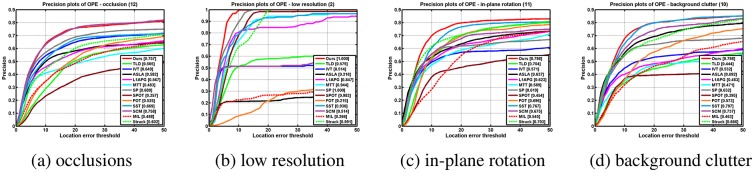
The plots of OPE with attributes based on the precision metric.

**Fig 8:**
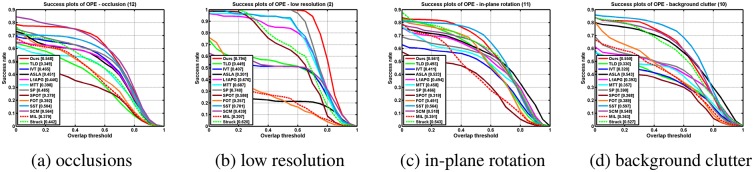
The plots of OPE with attributes using the success rate metric.

Table 1 shows the average overlap rates. Table 2 reports the average center location errors (in pixels) where a smaller average error means a more accurate result. Notice that the results are calculated by averaging 5 runs of these algorithms. As can be seen from the table, the most sequences generated by our method have lower average error and higher overlap rate values. We provide the precision and success plots in Fig 6 to evaluate our performance over all the 22 sequences. The evaluation parameters are set as default in [20]. We note that the our algorithm performs well for the videos with occlusion, low resolutionn, in plane rotation, and background clutter based on the precision metric and the success rate metric as shown in Figs 7 and 8 respectively. Both table and figures show that our method achieves favorable performance against other state-of-the-art methods.

**Table 1:** Average overlap rate and average frame per second (FPS). The best and the second results are shown in BOLD fonts and BOLD fonts, respectively.

	TLD	IVT	ASLA	*L*_1_APG	MTT	SP	SPOT	FOT	SST	SCM	MIL	Struck	Ours
Faceocc1	0.58	0.73	0.32	0.76	0.70	0.79	0.74	0.60	0.79	0.79	0.60	0.73	0.80
Faceocc2	0.62	0.73	0.65	0.69	0.75	0.59	0.69	0.64	0.63	0.73	0.67	0.79	0.69
David3	0.10	0.48	0.43	0.38	0.10	0.46	0.77	0.41	0.30	0.41	0.54	0.29	0.73
Boy	0.66	0.26	0.37	0.73	0.50	0.36	0.57	0.64	0.36	0.38	0.49	0.76	0.81
Deer	0.60	0.03	0.03	0.60	0.61	0.72	0.72	016	0.62	0.07	0.12	0.74	0.82
Fish	0.81	0.77	0.85	0.34	0.16	0.83	0.83	0.78	0.86	0.75	0.45	0.85	0.87
CarDark	0.45	0.66	0.85	0.88	0.83	0.77	0.00	0.26	0.86	0.84	0.20	0.89	0.85
Jogging-2	0.66	0.14	0.14	0.15	0.13	0.73	0.20	0.12	0.12	0.73	0.14	0.20	0.74
CarScale	0.45	0.63	0.61	0.50	0.49	0.60	0.01	0.35	0.55	0.59	0.41	0.41	0.81
Basketball	0.02	0.11	0.39	0.23	0.19	0.23	0.01	0.17	0.20	0.46	0.22	0.20	0.63
Football	0.49	0.56	0.53	0.55	0.58	0.69	0.01	0.55	0.40	0.49	0.59	0.53	0.59
Average	0.46	0.34	0.36	0.41	0.34	0.50	0.43	0.37	0.39	0.44	0.39	0.41	0.70
FPS	21.74	27.83	7.48	2.47	0.99	2.35	–	376.48	2.12	0.37	28.06	10.01	4.27

**Table 2:** Average center location error and average frame per second (FPS). The best and the second results are shown in BOLD fonts and BOLD fonts, respectively.

	TLD	IVT	ASLA	*L*_1_APG	MTT	SP	SPOT	FOT	SST	SCM	MIL	Struck	Ours
Faceocc1	27.37	18.42	78.06	17.33	21.00	14.14	17.17	29.00	13.00	13.04	29.86	18.78	12.88
Faceocc2	12.28	7.42	19.35	12.76	9.836	10.43	11.78	11.94	12.82	5.96	9.02	13.60	5.50
David3	208.00	51.95	87.76	90.00	341.33	8.74	6.27	33.40	104.50	73.09	29.68	106.50	5.79
Boy	4.49	91.25	106.07	7.03	12.77	58.09	8.93	5.79	66.97	51.02	12.83	3.84	2.57
Deer	30.93	182.69	160.06	24.19	18.91	6.84	13.95	80.30	13.81	103.54	100.73	5.27	4.59
Fish	6.54	5.67	3.85	29.43	45.50	3.99	4.52	6.50	3.14	8.54	24.14	3.40	3.08
CarDark	27.47	8.43	1.54	1.04	1.57	1.35	121.58	34.43	1.19	1.30	43.48	0.95	1.31
CarScale	22.60	11.90	24.64	79.78	87.61	13.36	207.01	106.20	87.05	33.38	33.47	36.43	7.66
Basketball	213.86	107.11	82.64	137.53	106.80	39.79	169.86	118.02	105.93	52.90	91.92	118.6	7.92
Football	14.26	14.34	15.00	15.11	13.67	5.22	202.03	13.36	17.21	16.30	12.09	17.31	7.28
Average	50.48	72.54	78.20	64.58	85.48	39.38	69.46	55.66	88.42	48.26	48.92	49.17	7.97
FPS	21.74	27.83	7.48	2.47	0.99	2.35	–	376.48	2.12	0.37	28.06	10.01	4.27

To further compare the running time of four subspace-based tracking algorithms (i.e. IVT, *L*_1_APG, SP and our method), we calculated the average Frames Per Second (FPS) for 32 × 32 image patch (see the last row of Table 1). For *L*_1_APG, we reported FPS for its APG acceleration. It can be seen that IVT is quite faster than other trackers as its computation only involves matrix-vector multiplication. Both SP and our method are faster than *L*_1_APG. It is also observed that our method is much faster than SP. This is due to the different choices of the optimization scheme. SP adopts a naive altering minimization strategy, in contrast, our method is efficiently solved by APG.

## Conclusion

In this paper, we propose sparse coding and counting method under Bayesian framwork for robust visual tracking. The proposed method combines *L*_0_ regularization and *L*_1_ regularized sparse representation in a unique one, therefore, it has better ability to sparsely represent an object and the reconstruction result are also better. Besides, to solve the proposed model, we develop a fast and efficient APG algorithm. Moreover, the closed solution of the combination of *L*_0_ norm and *L*_1_ norm regularization is provided. Extensive experiments testify to the superiority of our method over state-of-the-art methods, both qualitatively and quantitatively.
